# Recharacterization of EmAGA, a Potential Candidate for Novel ALL Therapeutics

**DOI:** 10.3390/biom16050690

**Published:** 2026-05-07

**Authors:** Qiange Lin, Yuxin Zhang, Junyou Lin, Yilin Ye, Xin Qian, Xinrong Lu, Shaoxian Lyu, Xinliu Geng, Li Chen, Guiqin Sun

**Affiliations:** 1 School of Medical Technology and Information Engineering, Zhejiang Chinese Medical University, Hangzhou 310053, China; 202311116011023@zcmu.edu.cn (Q.L.); 15858855885@alu.zcmu.edu.cn (Y.Z.); 202411126011010@zcmu.edu.cn (J.L.); 202311116011030@zcmu.edu.cn (Y.Y.); 202411126011013@zcmu.edu.cn (X.Q.); 2 Key Laboratory of Medical Molecular Virology, School of Basic Medical Sciences, Fudan University, Shanghai 200032, China; 20111010058@fudan.edu.cn (X.L.); 23111010078@m.fudan.edu.cn (S.L.); 25211010045@m.fudan.edu.cn (X.G.)

**Keywords:** aspartylglucosaminidase, acute lymphoblastic leukemia, asparagine, glutamine, autoproteolysis, molecular dynamics simulation

## Abstract

Aspartylglucosaminidase (AGA) is an amidohydrolase that can hydrolyze the amide bond between N-acetylglucosamine (GlcNAc) and asparagine (Asn), producing N-acetylglucosamine and aspartic acid (Asp). AGA is distributed in both eukaryotes and prokaryotes. In this study, we identified the sequence of AGA in *Elizabethkingia meningoseptica* (EmAGA), cloned and expressed it in *Escherichia coli*, and recharacterized its properties, confirming its substrates as aspartylglucosamine (Asn-GlcNAc) and Asn. Key residues affecting its enzymatic activity were predicted through molecular docking and conserved site analysis, and 10 key residues that affected enzymatic activity were verified, eight of which regulated activity by interfering with EmAGA’s autoproteolysis, indicating that autoproteolytic cleavage into α/β subunits was essential for EmAGA maturation. Molecular dynamics simulations were performed on autohydrolysis-impaired mutants, which showed a more stable conformation and lower energy. In summary, EmAGA’s functional characterization provided novel evidence for elucidating its molecular mechanism. Clinically used Asparaginase (ASNase) exerts its therapeutic effect on acute lymphoblastic leukemia (ALL) through its asparaginase activity, but it is limited by glutamine off-target side effects, while EmAGA also has asparaginase activity but no glutaminase activity, rendering its potential as a basis for novel anti-leukemic enzymatic therapeutics.

## 1. Introduction

Asparaginase (ASNase) is widely used in the treatment of acute lymphoblastic leukemia (ALL), and genetically engineered asparaginases from *Escherichia coli* and *Erwinia chrysanthemi* are the only formulations approved by the U.S. Food and Drug Administration (FDA) for clinical application [1,2,3]. Despite proven efficacy, these therapeutic enzymes exhibited an intrinsic affinity for glutamine (Gln), which constituted an off-target activity and thus induced adverse effects such as hepatotoxicity and hyperglycemia [4,5,6,7]. Earlier strategies to reduce off-target activity primarily involved genetic engineering of bacterial strains to screen mutants with reduced Gln affinity [8]. Thus, exploring low-toxic and highly specific asparaginase emerged as a research focus, with aspartylglucosaminidase possessing asparagine-related hydrolytic activity offering a promising direction.

Aspartylglucosaminidase (AGA, N4-(β-N-Acetylglucosaminyl)-L-Asparaginase, EC 3.5.1.26) is a widely distributed amidohydrolase [9,10]. It catalyzed the hydrolysis of the amide bond between *N*-acetylglucosamine (GlcNAc) and asparagine (Asn), producing *N*-acetylglucosamine and aspartic acid (Asp), and played a pivotal role in *N*-linked glycoprotein degradation [11,12]. In 1964, Murakami and Eylar first isolated AGA from sheep epididymis and named it β-(*N*-acetylglucosamine)-*N*-glycosidase [13]. To date, AGA has been purified or cloned from nearly 10 biological sources, and AGAs from diverse species share high sequence homology and conserved biochemical traits [14,15,16]. AGA, like all other members of the *N*-terminal nucleophile hydrolase family, is produced as a catalytically inactive precursor, and intramolecular autoproteolysis acts as the essential processing step required for its activation [17]. Specifically, after polypeptide chain translation, the nascent polypeptide chain sequesters the catalytic center via a surface loop (P-loop) [18], and cleavage of the P-loop is triggered by N → O acyl migration-mediated peptide bond rearrangement, which in turn generates the α and β subunits [19], ultimately generating active AGA with a heterotetramer (α_2_β_2_) structure [20]. However, the mechanism of polypeptide chain distortion-induced conformational changes during autoproteolysis, and the retention of local constraints during precursor folding, remained incompletely elucidated.

Previous studies had verified that the AGA from *Flavobacterium meningosepticum* (reclassified as *Elizabethkingia meningoseptica* in 2005) retained the AGA family’s inherent aspartylglucosamine (Asn-GlcNAc) activity and exhibited asparaginase activity, although its ability to hydrolyze Asn was only 9% of that toward Asn-GlcNAc [21]. Therefore, the present study aimed to further characterize the catalytic activity of AGA from *Elizabethkingia meningoseptica* (abbreviated as EmAGA), elucidate its regulatory mechanism of autoproteolysis, and identify the key amino acid residues critical for its autoproteolytic process and enzymatic activity. These investigations were anticipated to lay the foundation for EmAGA as a promising candidate to replace bacterial enzymes previously used in ALL therapy. Asparagine depletion was an effective strategy for ALL therapy because leukemic cells lacked asparagine synthetase and could not synthesize asparagine on their own. As an enzyme with asparaginase activity, EmAGA degraded asparagine in the microenvironment, thereby inhibiting the proliferation of leukemic cells.

## 2. Materials and Methods

### 2.1. Bioinformatics Analysis of EmAGA from Elizabethkingia meningoseptica

The DNA sequence of the putative *EmAGA* gene was derived from the whole-genome sequence of the clinical isolate (FMS-007) of *Elizabethkingia meningoseptica*. Protein sequences of EmAGA and its homologs from *Homo sapiens*, *Rattus norvegicus*, *Mus musculus*, *Macaca fascicularis*, *Bos taurus*, *Bombyx mori*, *Flavobacterium meningosepticum* and *Microscilla marina* were retrieved from the GenBank™ database [22]. The structures of wild-type EmAGA and its mutants were predicted online using AlphaFold3 (https://alphafoldserver.com/ (accessed on 22 January 2026)), and structural analyses were performed with PyMOL (2.5.7) software (https://www.pymol.org (accessed on 22 January 2026)). Multiple sequence alignment was carried out using online CLUSTALW (https://www.genome.jp/tools-bin/clustalw (accessed on 12 June 2025)), and alignment figures were generated via ESPript 3.0 software [23].

### 2.2. Cloning and Induced Protein Expression of EmAGA

The expression and purification of EmAGA and its mutant proteins were performed following established protocols with minor modifications. Briefly, sequence-verified recombinant EmAGA plasmids were transformed into *Escherichia coli* BL21 (DE3) competent cells (Solarbio, Shanghai, China). Transformed cells were inoculated into Luria–Bertani (LB) medium supplemented with 50 μg/mL kanamycin (Sangon Biotech, Shanghai, China) and cultured at 37 °C for 16 h to isolate single colonies. When the bacterial culture reached the logarithmic growth phase, isopropyl β-D-thiogalactoside was added to a final concentration of 1.0 mM, and the culture was incubated at 28 °C for 12 h to induce protein expression.

Bacterial cells were harvested by centrifugation at 2790× *g* for 10 min at room temperature, resuspended in pre-cooled lysis buffer (10 mM imidazole, 20 mM Tris-HCl, 500 mM NaCl, pH 7.4), and sonicated on ice for 30 min to disrupt cell membranes. The lysate was centrifuged at 15,909× *g* for 30 min at 4 °C to collect the supernatant. The supernatant was loaded onto a HisPur™ Ni-NTA Spin Column (Thermo Scientific, Waltham, MA, USA) and incubated for 30 min to allow binding of the His-tagged target protein. The column was sequentially washed five times with lysis buffer and five times with wash buffer (25 mM imidazole, 20 mM Tris, 500 mM NaCl, pH 7.4) to remove impurities, and the target protein was eluted with elution buffer (250 mM imidazole, 20 mM Tris, 500 mM NaCl, pH 7.4). Purified EmAGA protein was concentrated using an Amicon Ultra 3K Ultrafiltration Unit (Millipore, Billerica, MA, USA). The same protocol was applied for the expression and purification of all EmAGA mutant proteins. A 2 μL aliquot of the purified EmAGA and its mutant proteins was subjected to absorbance measurement at 280 nm using the Protein A280 mode on a NanoDrop™ One/One C Microvolume UV–Vis Spectrophotometer (Thermo Scientific, Waltham, MA, USA). Protein concentration was calculated using the theoretical extinction coefficient derived from the amino acid sequence of EmAGA via the online ProtParam tool (https://web.expasy.org/protparam/ (accessed on 11 March 2026)): Abs_0.1_% (=1 g/L) = 0.964. The calculation formula was as follows: concentration (mg/mL) = A_280_/0.964.

### 2.3. Activity Assay with Asp(pNA)-OH

The reaction system (100 μL total volume) contained 2 μg of EmAGA (final concentration: 0.02 mg/mL), 1 mM Aspartic Acid β-(*p*-nitroanilide) (abbreviated as Asp(pNA)-OH), and PBS (8 mM Na_2_HPO_4_, 136 mM NaCl, 2 mM KH_2_PO_4_, 2.6 mM KCl, pH 7.4) buffer. The mixture was incubated statically at 37 °C for 2 h in a 96-well plate, and the release of *p*-nitroaniline was detected by measuring absorbance at 405 nm (*ε*_405_ = 9920 M^−1^cm^−1^) using BioTek Epoch 2 microplate spectrophotometer (BioTek, Winooski, VT, USA) [15,24]. The absorbance in this experiment was only used as a reference for the color development trend. The corresponding concentration-gradient standard curve was provided in Figure S1 to show the chromogenic response of the substrate. All enzymatic activity assays in this study were performed in three independent replicates (*n* = 3). Data were presented as mean ± standard deviation (SD), with error bars shown in the figures.

### 2.4. Effects of Reaction Conditions on EmAGA Activity

Enzyme activity was measured using Asp(pNA)-OH as the substrate according to the assay procedure described in Section 2.3.

The reaction systems were pre-incubated at 25, 35, 45, 50, 55, 65, and 75 °C for 30 min, respectively. After adding EmAGA, the mixtures were incubated for a further 2 h.

To investigate the effect of pH on EmAGA activity, the enzyme activity was measured in citrate–sodium citrate buffer (100 mM, pH 4–5), phosphate buffer (100 mM, pH 6–8), and borax–boric acid buffer (50 mM, pH 9–11).

The effects of metal ions were determined by adding MgCl_2_, CaCl_2_, ZnSO_4_, and BaCl_2_ to final concentrations of 0, 1, 10, 25, 50, 75 mM, and reactions were incubated at 37 °C for 2 h.

The effects of amino acids were evaluated by supplementing the reaction system with glycine, glutamine and alanine at final concentrations of 0, 1, 5, 10, 20, 50, 100, 150 mM, followed by incubation at 37 °C for 2 h.

### 2.5. Activity Assay with Asn-GlcNAc and Peptide-GlcNAc (GlcNAc Release Detection)

The reaction mixture consisted of 20 μL of 20 mM sodium phosphate buffer (pH 7.5) containing 2.5 mM Asn-GlcNAc and 4 μg of EmAGA. After the mixed system was added into an EP tube and incubated in a water bath at 37 °C for 2 h, the reaction was terminated by adding 50 μL of 250 mM sodium borate buffer (pH 8.8) followed by boiling at 100 °C for 3 min. Released *N*-acetylglucosamine (GlcNAc) was quantified via the Morgan–Elson reaction at 530 nm using a total amino sugar assay kit (Genmed, Shanghai, China). In the reaction, GlcNAc was heated under alkaline conditions. Subsequently, 50 μL of Ehrlich’s reagent (acidic *p*-dimethylaminobenzaldehyde) was added, and the mixture was incubated at room temperature in the dark for 10 min to form a purple-red complex. The absorbance was measured at 530 nm using BioTek Epoch 2 microplate spectrophotometer (BioTek, Winooski, VT, USA) [25].

### 2.6. Activity Assay with Glutamine and Asparagine (Aspartate Release Detection)

The reaction system (20 μL total volume) included 10 μL of 40 mM substrate, 5 μL of EmAGA, and PBS buffer. After water bath incubation at 37 °C for 2 h, 80 μL of working solution prepared from the Amplex Red Aspartate Assay Kit (Beyotime Biotechnology, Beijing, China) (72 μL Aspartate Assay Buffer, 2 μL Amplex Red, 2 μL Enzyme Solution A, 2 μL Enzyme Solution B, 2 μL Cofactor) was added to the 96-well plate. The mixture was vortexed gently and statically incubated at 37 °C for 30 min in the dark, and aspartate release was measured at 570 nm (*ε*_570_ = 54,000 M^−1^cm^−1^) using BioTek Epoch 2 microplate spectrophotometer (BioTek, Winooski, VT, USA) [26].

### 2.7. Model of EmAGA–Ligand Recognition

EmAGA was modeled using AlphaFold3 (https://alphafoldserver.com/ (accessed on 22 January 2026)) [27]. Molecular docking between EmAGA and its substrate was performed using the Schrödinger (12.8.117) software (https://www.schrodinger.com (accessed on 29 January 2026)). Detailed methods are described as follows. Hydrogen atoms were added and missing residues were restored using the Protein Preparation Wizard in Schrödinger. Asn-GlcNAc and Asn were selected as the ligand; their 3D structure were retrieved from PubChem, protonated at physiological pH, and energy-minimized via LigPrep module.

The highest-ranked active site identified by the pocket-detection algorithm was used to define the docking box. A receptor grid centered at (x = −2.65, y = −0.62, z = −14.5) was generated, encompassing catalytic residues within a 10 Å sphere of the binding pocket. Semi-flexible docking of Asn-GlcNAc and Asn were performed in internal-coordinate space, and virtual screening of EmAGA–ligand complexes was carried out using Glide SP.

### 2.8. Molecular Dynamics Simulation and Analysis of EmAGA

Structures of EmAGA mutants (W11A, E48A, T152A, T169A, G172A, R180A, G204A, Q254A) were predicted using AlphaFold3. Molecular dynamics simulations were performed with GROMACS (2022.2 version) using the CHARMM36 force field (2022 version) for both wild-type and mutant EmAGA systems [28,29].

### 2.9. Simulation Protocol

Energy minimization: Ion-added systems were pre-minimized, followed by steepest descent energy minimization (energy threshold: 1000.0 kJ/mol/nm).

Equilibration: NVT ensemble: 100 ps (0.1 ns) at 300 K, with V-rescale temperature coupling and constraints on protein backbone/side chains. NPT ensemble: 100 ps (0.1 ns) at 1 bar, with isotropic Parrinello–Rahman pressure coupling.

Production run: 200 ns simulation at 300 K and 1 bar, with LINCS algorithm for hydrogen bond constraints, V-rescale temperature coupling (T = 0.1 ps), Parrinello–Rahman pressure coupling (T = 2.0 ps), 2 fs integration time step, and Verlet cutoff scheme (non-bonded interactions truncated at 1.0 nm). Long-range electrostatic interactions were calculated using the Particle Mesh Ewald (PME) method.

### 2.10. Trajectory Analysis

Root mean square deviation (RMSD), root mean square fluctuation (RMSF), solvent-accessible surface area (SASA), radius of gyration (Rg), intramolecular hydrogen bonds, and Gibbs free energy were analyzed using built-in GROMACS (2022.2 version) modules.

## 3. Results

### 3.1. Predictive Aspartylglucosaminidase from Elizabethkingia meningoseptica FMS007

The whole-genome sequence of *Elizabethkingia meningoseptica* FMS-007 was completed in our laboratory in 2015, yielding a complete sequence of 3,938,967 base pairs (GenBank: CP006576) [30]. Through bioinformatics analysis, a 996 bp open reading frame (ORF) was identified and annotated as a predicted AGA encoding 331 amino acids with a 36-amino acid signal peptide, named EmAGA. EmAGA and human AGA have a high sequence homology with an E-value of 1 × 10^−59^ in BLAST analysis.

### 3.2. Enzyme Activity Analysis of EmAGA

**Figure 1:**
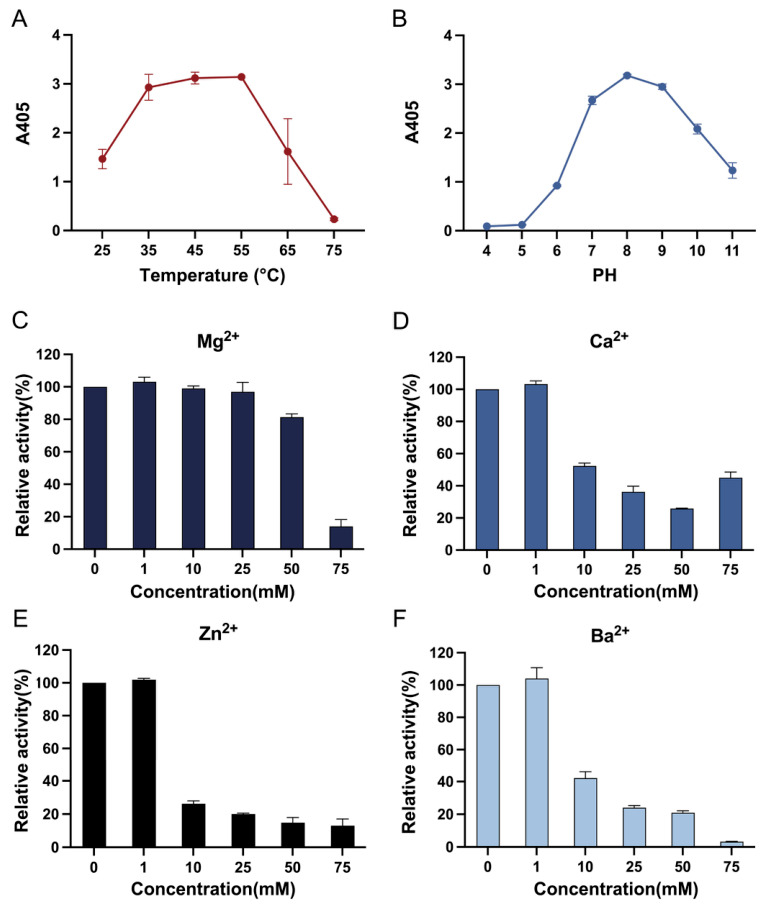
Enzymatic characterization of EmAGA. (**A**) Optimal temperature of EmAGA. (**B**) Optimal pH on EmAGA activity. (**C**–**F**) Effect of metal ions on EmAGA activity.

The EmAGA derived from *Elizabethkingia meningoseptica* obtained above was subjected to gene cloning, expression, and purification in the *E. coli* BL21 (DE3) expression system. To characterize the enzymatic activity of EmAGA, the substrate Asp(pNA)-OH (a commonly used substrate for AGA assays) was employed in this study. The enzyme exhibited high activity between 35 °C and 55 °C, while the activity at 25 °C and 65 °C was only approximately 50% of the maximum activity (Figure 1A). AGA from various species, including human, rat, pig, cattle, and mice, generally possesses a relatively high optimal reaction temperature and maintains stability at 60 °C [15]. Similar to AGA from other species, this enzyme exhibited activity over a broad pH range of 6–11, with an optimal pH of 8 (Figure 1B). Metal ions were added to the reaction system at final concentrations ranging from 0 to 75 mM. Ca^2+^, Ba^2+^ and Zn^2+^ exhibited strong inhibitory effects on EmAGA activity at a concentration of 10 mM, whereas Mg^2+^ only exerted a potent inhibitory effect at 75 mM (Figure 1C–F). Notably, the apparent recovery of enzyme activity observed at 75 mM Ca^2+^ was caused by slight precipitation at high calcium concentrations, which interfered with absorbance detection and led to artificially elevated activity values.

### 3.3. Substrate Specificity and Effects of Amino Acids on EmAGA

**Figure 2:**
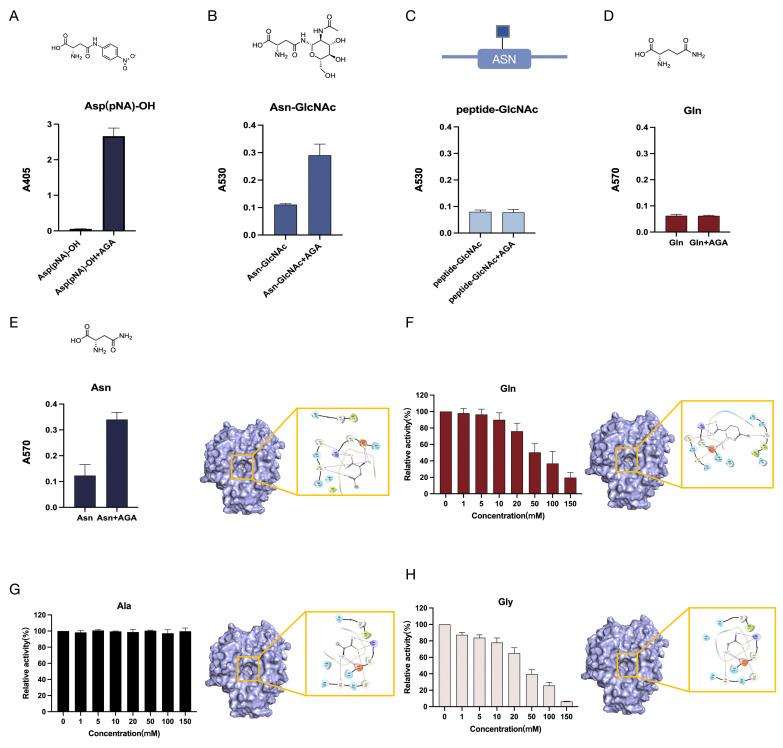
Substrate specificity of EmAGA and effects of amino acids on its activity. (**A**) Aspartic Acid β-(*p*-nitroanilide) (Asp(pNA)-OH). (**B**) aspartylglucosamine (Asn-GlcNAc). (**C**) peptide-GlcNAc. (**D**) Gln. (**E**) asparagine (Asn) and its molecular docking of amino acids with EmAGA. (**F**–**H**) Effect of amino acids on EmAGA activity and its molecular docking of amino acids with EmAGA. Hydrogen bonds were depicted as purple arrows, and salt bridges were presented as purple-red lines. Residues were colored by type: hydrophobic residues in green, positively charged residues in purple, negatively charged residues in orange, polar residues in light blue, and glycine residues in pale off-white. Black lines represented the protein backbone trace.

In this study, the substrate preference of EmAGA was determined. Specifically, EmAGA could efficiently hydrolyze Asp(pNA)-OH, Asn-GlcNAc, and Asn (Figure 2A,B,E). By contrast, it displayed no hydrolytic effect on peptides-GlcNAc (products from Endo F-mediated digestion of RNase B), and Gln (Figure 2C,D). Overall, EmAGA exhibited clear selectivity toward various substrates. Based on the molecular docking model, this study further elucidated the structural mechanism by which EmAGA preferentially bound Asn rather than Gln. The substrate binding region of EmAGA adopted an overall compact structure. Asn possessed a shorter side chain and showed better spatial compatibility. It was precisely anchored inside the binding region and formed stable interactions with key residues Arg-180, Asp-183, Gly-204 and Gly-206 through its backbone and side-chain groups. In comparison, Gln had one extra methylene group, which resulted in a longer overall extended side chain. Restricted by the narrow and compact binding cavity, the additional elongated side chain fragment of Gln failed to form effective interactions with surrounding amino acid residues. We therefore speculated that this structural mismatch might cause Gln to exhibit weaker overall anchoring and binding ability than Asn.

Further investigation into the effects of amino acids on EmAGA activity was conducted. High concentrations of Gly and Gln exerted potent inhibitory effects on EmAGA activity, whereas Ala had no impact on its activity. Molecular docking analysis of the binding interactions between EmAGA and these amino acids revealed that Gly, Gln, and Ala were all capable of binding to the active pocket of EmAGA. Mechanistically, Gly and Gln inhibited EmAGA activity through competitive binding to the active region. Gly was smaller in molecular size than Ala, its side chain containing only hydrogen atoms could smoothly enter the active region and achieve stable binding. We speculated that the methyl group of Ala caused unfavorable steric hindrance in the spatially confined active site, preventing stable binding to the enzyme and resulting in no inhibitory activity. In summary, EmAGA can hydrolyze Asn but exhibits no hydrolytic activity toward Gln.

In the present study, we compared the substrate specificities of EmAGA, the previously reported AGA from Homo sapiens, and ASNase used for the treatment of ALL (Table 1). The results showed that both EmAGA and human AGA could catalyze the hydrolysis of Asn-GlcNAc and L-Asn, whereas ASNases from *Escherichia coli* and *Erwinia chrysanthemi* exhibited catalytic activity toward L-Asn, D-Asn, and Gln [31,32,33,34].

### 3.4. Amino Acid Residues Affecting EmAGA Enzymatic Activity

**Figure 3:**
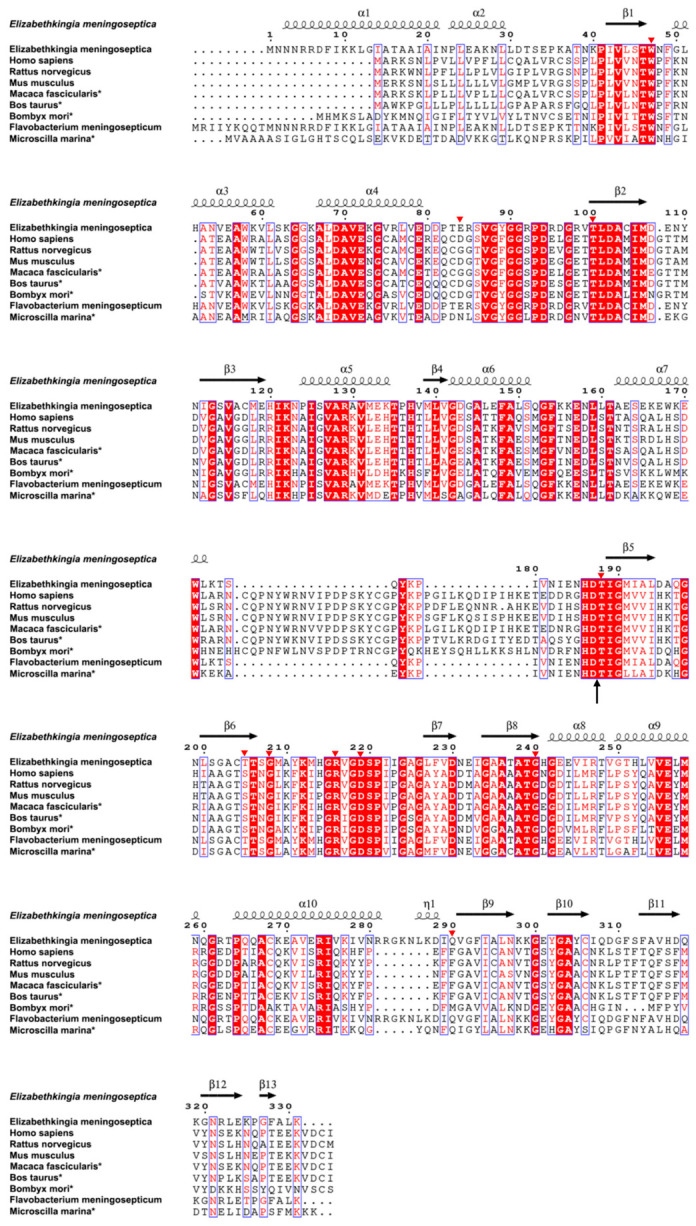
Sequence analysis of AGA. Structure-based sequence alignment of AGA (including signal peptide). The amino acid sequence of AGA of *Elizabethkingia meningoseptica* (GenBank: CP006576), *Flavobacterium meningosepticum* (GenBank: Q47898.1), *Microscilla marina* (GenBank: EAY27886.1), *Rattus norvegicus* (GenBank: P30919.2), *Homo sapiens* (GenBank: NP_000018.1), Mus musculus (GenBank: Q64191.1), *Macaca fascicularis* (GenBank: Q4R6C4.1), *Bos taurus* (GenBank: XP_005226002.1), and *Bombyx mori* (GenBank: NP_001037686.1) were retrieved from the GenBank™ database [22]. Amino acid sequences deduced from whole genome sequencing were marked with an asterisk (*) after the species names. Residues in white on red background are evolutionarily conserved key sites identified by sequence analysis. All amino acid mutation sites constructed in this experiment were marked with red triangles. The autoproteolytic cleavage site between Asp-151 (187, full-length including the 36-residue signal peptide) and Thr-152 (188, full-length including the 36-residue signal peptide) was marked with an arrow.

**Figure 4:**
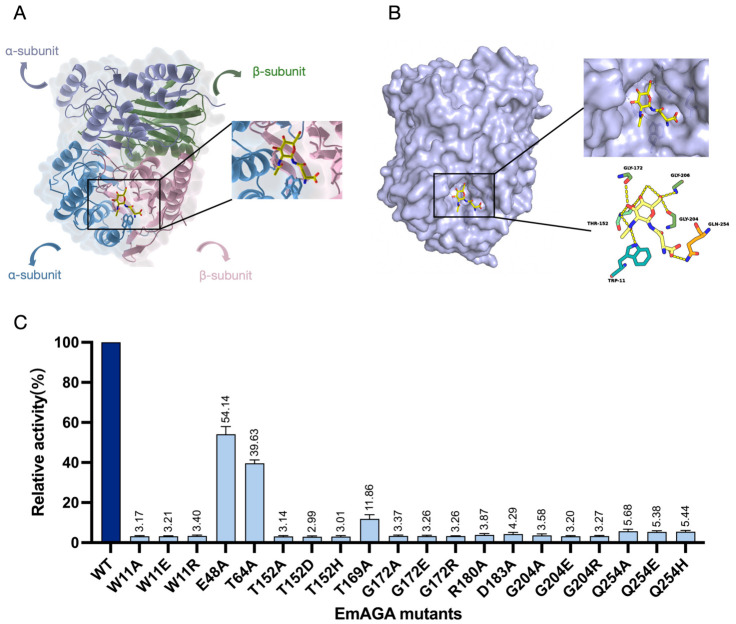
Amino acid residues affecting EmAGA activity. (**A**) Three-dimensional structure prediction of EmAGA. EmAGA adopted an αββα tetramer fold. The α-subunits were shown in blue and purple, and the β-subunits were rendered in pink and green, respectively. The (**left**) panel displays the overall prediction structure, and the (**right**) panel shows the locally magnified image. (**B**) Schematic diagram of the docking between EmAGA and Asn-GlcNAc molecules. The (**left**) panel presents the overall prediction result in surface representation, and the (**right**) panel shows the locally magnified image. (**C**) Relative enzymatic activity of EmAGA and its mutants.

To identify the key amino acid residues of EmAGA, a structure-based sequence alignment was performed between EmAGA and the previously reported and predicted AGA sequences in this study (Figure 3). Among the AGAs from humans, animals, bacteria, and other sources, 85 consensus residues were identified within the over 300-amino acid sequence, including the autohydrolytic cleavage site between Asp-151 and Thr-152 (corresponding to Asp-187 and Thr-188 in the full-length sequence with the 36-residue signal peptide; Figure 3), which is essential for the activation of inactive nascent peptides into mature active AGA enzymes. Using AlphaFold3 to predict the three-dimensional structure of EmAGA, both the α-subunit and β-subunit together formed a four-layer αββα structure (Figure 4A), with two β-sheets stacked against each other to form a core that was surrounded on the outside by two layers of α-helices. The eight β-strands from the four strands from the α-subunit and four strands from the β-subunit formed the first β-sheet, while another β-sheet was composed of four antiparallel β-strands from the β-subunit. The α-helical layer outside the eight β-strands was formed by five α-helices that belonged to the α-subunit, and another α-helical layer outside the four β-strands was connected by three α-helices from the β-subunit.

Molecular docking simulations were performed using the predicted structure of EmAGA with the substrates Asn-GlcNAc and Asn. The constructed docking model was validated via structural alignment with the published crystal structure of AGA-substrate complex from *Elizabethkingia meningoseptica* (PDB: 4R4Y). The results demonstrated that the substrate binding regions of the two structures were highly consistent, verifying the authenticity and reliability of the present molecular docking model (Figure S2). The results suggested that Trp-11, Thr-152, Gly-172, Arg-180, Asp-183, Gly-204, Gly-206 and Gln-254 were potential key residues regulating the enzymatic activity of EmAGA (Figure 4B). Among the eight binding sites obtained by molecular docking, all were highly conserved except for Gln-254. Based on the amino acid residues predicted by molecular docking simulations, combined with three residues previously reported in the literature, site-directed mutagenesis was performed on a total of 10 amino acids (all mutated residues were clearly labeled as red triangles in Figure 3) [35]. Using in vitro site-directed mutagenesis technology, 20 distinct active site substitution mutations were introduced into the *AGA* gene, specifically including: W11A, W11E, W11R, E48A, T64A, T152A, T152D, T152H, T169A, G172A, G172E, G172R, R180A, D183A, G204A, G204E, G204R, Q254A, Q254E, and Q254H.

After the completion of autoproteolysis of the mutant precursors, the activities of the wild-type (WT) AGA and each mutant were determined via a colorimetric assay using 1 mM Asp(pNA)-OH as the substrate (Figure 4C). The enzymatic activities of all mutants were significantly reduced, with the degree of activity impairment varying remarkably among different site mutations. Notably, the enzymatic activities of 14 mutants were directly reduced to the background level. Even for E48A and T64A, the two mutants with relatively higher residual activity, their enzymatic activities were only maintained at 54.14% and 39.63% of the WT, respectively.

### 3.5. Analysis of EmAGA Autoproteolysis

**Figure 5:**
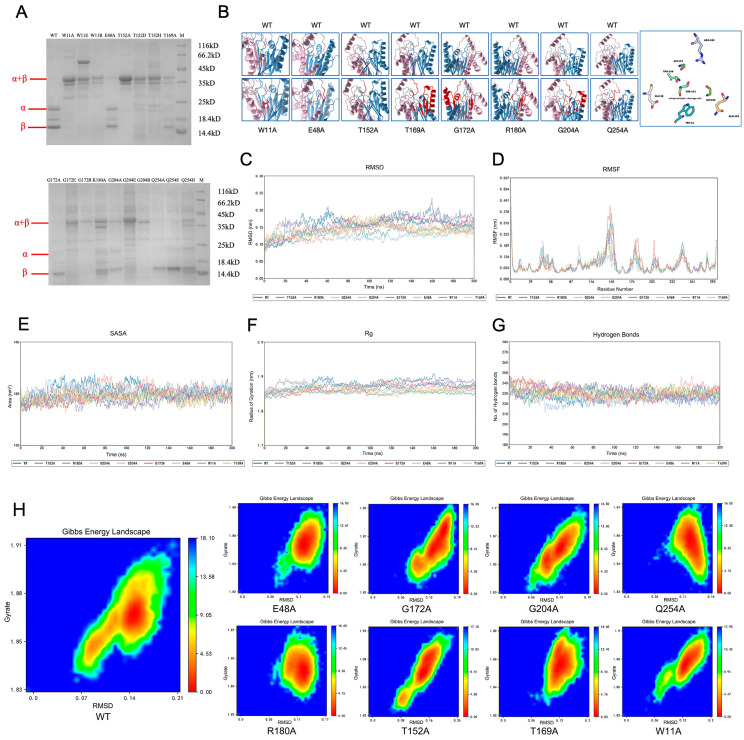
Preliminary mechanism affecting autohydrolysis. (**A**) SDS-PAGE analysis of autohydrolysis-related mutants. The (**upper**) and (**lower**) panels show the autohydrolysis profiles of wild-type (WT) EmAGA and its mutants. Bands corresponding to the full-length α+β precursor, the cleaved α-subunit, and the cleaved β-subunit are indicated. (**B**) Three-dimensional structural comparison of EmAGA mutants. The (**upper**) row displays the overall structure of WT EmAGA. The (**lower**) row shows local conformational changes at the autoproteolytic cleavage site Thr-152 in each mutant. Regions showing conformational differences from the WT structure are marked in red. The rightmost panel shows the spatial distribution of key amino acid residues related to the autoproteolysis of EmAGA in surface representation. (**C**) Root mean square deviation (RMSD) plots of EmAGA and its mutants relative to the initial structure, reflecting the overall structural stability of each mutant. The *X*-axis depicts the simulation time (ns), whereas the *Y*-axis depicts the RMSD value (nm). (**D**) Root mean square fluctuation (RMSF) plots of EmAGA and its mutants, showing the flexibility of each residue along the polypeptide chain. Peaks indicate regions with high conformational dynamics. The *X*-axis depicts the residue number, whereas the *Y*-axis depicts the RMSF value (nm). (**E**) Solvent-accessible surface area (SASA) plots of EmAGA and its mutants, reflecting the overall exposure of the protein to solvent. The *X*-axis depicts the simulation time (ns), whereas the *Y*-axis depicts the SASA value (nm^2^). (**F**) Radius of gyration (Rg) plots of EmAGA and its mutants, indicating the compactness of the overall protein structure. The *X*-axis depicts the simulation time (ns), whereas the *Y*-axis depicts the Rg value (nm). (**G**) Time evolution plots of the number of intramolecular hydrogen bonds in EmAGA and its mutants, reflecting the stability of the protein hydrogen-bond network. The *X*-axis depicts the simulation time (ns), whereas the *Y*-axis depicts the number of hydrogen bonds. (**H**) Gibbs free energy landscapes of EmAGA and its mutants. The color bar represents the free energy value (kJ/mol), with red/orange indicating low-energy, stable conformational states and blue indicating high-energy, unstable states.

Analysis of the SDS-PAGE electrophoresis results revealed that, among the aforementioned mutants with significantly reduced enzymatic activity, the impaired enzymatic activity of the mutants harboring mutations at eight distinct sites was attributed to abnormal autoproteolysis (Figure 5A). WT EmAGA exhibited complete autoproteolytic activity, with distinct and well-resolved bands corresponding to the α-subunit and β-subunit observed on SDS-PAGE. In contrast, all the tested mutants displayed incomplete or defective initial autoproteolysis, which was characterized by the presence of an uncleaved precursor protein band (α+β) at approximately 39 kDa. Mutations at the aforementioned sites directly abrogate the autoproteolytic process of EmAGA, and also identified Trp-11, Glu-48, Thr-152, Thr-169, Gly-172, Arg-180, Gly-204 and Gln-254 as the key active sites governing EmAGA autoproteolysis. These results demonstrate that autoproteolysis is an indispensable prerequisite for EmAGA to form its mature and active conformation.

To elucidate the structural basis for its autoproteolysis deficiency, the spatial conformation of the three-dimensional structure of EmAGA was analyzed in this study. The simulated structures of wild-type EmAGA and mutated EmAGA predicted by AlphaFold3 were presented in Figure 5B. Thr-152 was located in the loop region at the junction of α-subunit and β-subunit and represented the exact site of autoproteolytic cleavage. Structural analysis revealed that mutation of Thr-152, the canonical autoproteolytic cleavage site of EmAGA, to alanine (T152A) induced a prominent conformational alteration in this region, which directly impaired the progression of the autoproteolytic process. Following the substitution of Trp-11 with alanine (W11A), an α-helical segment within this region underwent a rotational change of approximately 90°. Alanine substitutions at Thr-169, Gly-172, Arg-180 and Gly-204 induced angular deflection of surrounding α-helix and β-sheet elements. The β-sheet segments of G172A and R180A underwent a conformational transition from downward arrangement to upward tilting, which further caused positional deviation of the autoproteolytic loop containing the Thr-152 site. E48A and Q254A presented limited structural perturbation and only resulted in subtle local displacement of the loop region (Figure 5B). Although Trp-11, Glu-48, Arg-180, Gly-204 and Gln-254 are spatially distant from the Thr-152 cleavage site in the primary protein sequence, the spatial distribution of key residues of EmAGA confirmed that these residues are all localized in the proximal region of the EmAGA autoproteolytic active center (Figure 5B). Alanine substitution of these residues indirectly perturbed the spatial conformation of the autoproteolytic cleavage site, thereby interfering with the autoproteolytic process.

### 3.6. Analysis of the Autoproteolysis Mechanism

The root mean square deviation (RMSD) analysis indicated that both WT and mutant of EmAGA proteins reached a stable state in the late stage of the simulation, suggesting that the simulation systems had achieved a relatively balanced state (Figure 5C). Based on the fluctuation data, the higher root mean square fluctuation (RMSF) value of the WT indicated that it possessed better structural flexibility than all the mutants (Table 2). Specifically, the average RMSF value of WT EmAGA was 0.083 nm. The G172A mutant exhibited an identical average RMSF value to the WT, while all other mutants showed slightly lower average RMSF values ranging from 0.066 nm to 0.082 nm. Although the overall numerical differences were relatively modest, the vast majority of mutants displayed a consistent downward trend. Notably, both the WT and all mutants exhibited significantly higher fluctuations in the amino acid region of 140–150 (a loop adjacent to the autoproteolytic cleavage site) compared with other regions, suggesting a more vigorous motion of this fragment (Figure 5D). A lower RMSF value in the mutants compared to WT reflects a reduction in protein structural flexibility, which demonstrated that the aforementioned mutations imposed a restrictive effect on protein flexibility. The decreased overall flexibility of the mutants would hinder the conformational distortion of the scissile peptide bond required for autoproteolysis. The radius of gyration (Rg) values of all mutants (1.854–1.879 nm) were close to that of the WT, indicating that the mutations only affected the local conformation of EmAGA without altering its overall folded structure (Figure 5F).

The solvent-accessible surface area (SASA) values of most mutants were slightly lower than that of WT EmAGA, with only G204A showing a SASA value close to the WT level (Table 2, Figure 5E). SASA reflects the extent of interaction between the protein surface and the solvent. The reduced SASA values of the mutants indicated a more compact protein conformation and weakened protein–solvent surface interactions. This might lead to insufficient exposure of the key functional groups in the active center (e.g., the hydroxyl group of Thr152), thereby impairing autoproteolytic function. The average number of intramolecular hydrogen bonds in most mutants was slightly higher than that in WT EmAGA (Table 2), suggesting that the mutants enhanced their structural compactness and stability through the formation of additional intramolecular hydrogen bonds (Figure 5G). In contrast, E48A exhibited a lower number of intramolecular hydrogen bonds, which was consistent with its residual autoproteolytic activity and an enzymatic activity of 54.14% relative to the WT.

In addition, the Gibbs free energy of all atoms in the WT and mutant EmAGA systems was calculated from the simulation trajectories to determine the tendency of conformational fluctuations. The results showed that the Gibbs free energy change (ΔG) of the WT was higher than those of all the mutants (Table 2, Figure 5H). Compared with the mutants, the low-energy regions of WT EmAGA were more dispersed, indicating that the WT had higher conformational energy and lower structural stability.

In summary, the key amino acid residues governing the autoproteolysis and enzymatic activity of EmAGA are distributed on both the α and β subunits, with a predominant enrichment on the β subunit (Table 3). The α subunits and β subunits can synergistically regulate the autoproteolysis and catalytic activity of EmAGA, and the spatial structural assembly between the subunits is critical for maintaining the normal function of the enzyme. The core regulatory role of the β subunit in EmAGA autoproteolysis and enzymatic activity provides a clear target for the precise molecular modification of this enzyme. This study elucidates the molecular mechanism underlying the functional regulation of EmAGA and lays a theoretical foundation for its directed evolution and the improvement of its application potential in industrial biocatalysis.

## 4. Discussion

As a class of amide hydrolases, AGA was widely distributed in organisms, with homologous sequences identified across prokaryotes and eukaryotes. In mammals, AGA had been successfully detected in the tissues of vertebrates including humans, pigs, cattle, rats and mice [15]. As a key functional enzyme, it participated in the ordered degradation of N-linked glycoproteins. Deficiencies in the function of human AGA lead to aspartylglucosaminuria (AGU), a hereditary lysosomal storage disease that causes severe multisystem physiological damage to the body [36,37,38,39]. In insects, AGA from parasitic wasp venom had been confirmed to act as a virulence factor involved in the regulation of host physiology [16].

The AGA family shared common three-dimensional structural characteristics, possessing a conserved αββα sandwich fold [40]. Furthermore, the nascent single-chain precursor of all AGA members required self-cleavage to generate α and β subunits, a process that was essential for the formation of active heterotetramers and the acquisition of catalytic activity. However, significant differences existed in the mechanisms maintaining structural stability among AGA family members derived from different sources. For eukaryotic AGA from mammals, insects and other taxa, structural stability depended on the maintenance of intersubunit disulfide bonds. Taking human AGA as a representative example, its molecule contained four pairs of disulfide bonds, and the C-terminus of its α subunit required proteolytic truncation of 10–20 amino acid residues to complete the maturation process [41]. In contrast, EmAGA, a bacterial homolog, lacked disulfide bonds and exhibited a more streamlined overall structure, which indicated that distinct mechanisms were employed by the AGA family to maintain structural stability [15]. In this study, molecular docking and activity assays were performed to verify the residues affecting enzymatic activity. Q254 was newly identified as a critical residue regulating autoproteolysis. Mutation at this site blocks autoproteolysis, hinders the formation of the mature enzyme, and consequently leads to reduced overall catalytic activity. Additionally, SDS-PAGE analysis was conducted to confirm the key residues involved in modulating EmAGA autoproteolytic. According to the previously reported “distortion-breakage” hypothesis proposed by Sui, the initiation of AGA autoproteolytic required a high-energy distorted conformation adjacent to the scissile peptide bond, which destabilized the precursor ground state and thereby reduced the activation energy barrier for N-O acyl transfer [18]. In the present work, molecular docking and molecular dynamics simulations were utilized to elucidate the mechanism by which active site residues regulated EmAGA autoproteolytic. The results revealed that the primary factor influencing the autoproteolytic capacity of EmAGA mutants was the alteration of local amino acid spatial conformation. In general, the reduced autoproteolytic ability of the mutants may be associated with their lower Gibbs free energy, increased number of hydrogen bonds, and decreased structural flexibility. These conformational changes eliminated the conformational tension necessary for autoproteolytic, resulting in a significant reduction in autoproteolytic activity.

Based on its unique substrate specificity, EmAGA exhibited a promising application prospect. It showed potential in the clinical treatment of ALL. ASNase, a conventional therapeutic agent for ALL, had certain limitations in clinical application [42,43,44,45]. ASNase possesses dual Asn and Gln activities, and its off-target Gly activity induced severe adverse effects such as hepatotoxicity and neurotoxicity [46,47,48,49,50]. In comparison, EmAGA displayed strict substrate specificity for Asn and completely lacked glutaminase activity, thus providing a novel alternative strategy to address the side effects associated with ASNase therapy.

The enzymatic functions of EmAGA have been characterized to date, whereas its biological functions remain elusive. To date, besides its identification in *Elizabethkingia meningoseptica*, AGA may also be present in other prokaryotes, and the biological functions of AGA in both prokaryotic and eukaryotic organisms await further in-depth investigation.

## 5. Conclusions

In conclusion, we identified an aspartylglucosaminidase (EmAGA) from *Elizabethkingia meningoseptica* and recharacterized its structural and enzymatic properties. Key residues affecting its enzymatic activity and autoproteolysis were identified, and we revealed the molecular mechanism of conformational changes during EmAGA autoproteolysis through molecular dynamics simulations. More importantly, EmAGA specifically acts on Asn without glutaminase off-target activity, showing great potential as a novel anti-leukemic enzymatic therapeutic agent.

## Figures and Tables

**Table 1:** Comparison of AGA and Asparaginase (ASNase).

Enzyme	Asn- GlcNAc	Peptide-GlcNAc	L-Asn	D-Asn	Gln
EmAGA	+	−	+	/	−
AGA from *Homo sapiens*	+	/	+	/	−
ASNase from *Escherichia coli*	/	/	+	+	+
ASNase from *Erwinia chrysanthemi*	/	/	+	+	+

+: Hydrolyzed by the enzyme; −: Not hydrolyzed by the enzyme. /: Not tested.

**Table 2:** The Gibbs free energy, RMSD, RMSF, Rg, SASA, and hydrogen bonds generated in phosphorylated and AGA mutant were compared on an average basis.

Protein	Gibbs Free Energy (kJ/mol)	Hydrogen Bonds	SASA (nm^2^)	RMSF (nm)	RMSD (nm)	Rg (nm)
WT	18.1	227.606	150.735	0.083	0.153	1.872
E48A	16.9	223.565	150.734	0.066	0.127	1.86
T152A	17.1	230.243	149.201	0.082	0.165	1.876
T169A	16.6	230.730	149.050	0.078	0.147	1.865
G172A	16.3	230.005	149.584	0.083	0.154	1.866
R180A	16.6	230.394	149.748	0.073	0.134	1.854
Q254A	16.5	229.008	149.399	0.070	0.137	1.858
W11A	17.9	231.125	148.659	0.078	0.161	1.879
G204A	16.8	232.176	150.634	0.081	0.138	1.865

**Table 3:** Amino acids affecting the autohydrolysis and enzymatic activity of EmAGA.

Subunit	Mutation	Activity (%)	Autohydrolysis	Precursor	α	β
α-subunit	WT	100	N	−	+	+
	W11A	3.17	Y	+	+	+
	W11E	3.21	Y	+	+	+
	W11R	3.30	Y	+	+	+
	E48A	54.14	Y	+	+	+
	T64A	39.63	N	−	+	+
β-subunit	T152A	3.14	Y	+	−	−
	T152D	2.99	Y	+	+	+
	T152H	3.01	Y	+	+	+
	T169A	11.86	Y	+	+	+
	G172A	3.37	Y	+	+	+
	G172E	3.26	Y	+	+	+
	G172R	3.26	Y	+	+	+
	R180A	3.87	Y	+	+	+
	D183A	4.29	N	−	+	+
	G204A	3.58	Y	+	+	+
	G204E	3.20	Y	+	+	+
	G204R	3.27	Y	+	−	−
	Q254A	5.68	Y	+	+	+
	Q254E	5.38	Y	+	+	+
	Q254H	5.44	Y	+	+	+

Y: Partial or complete effect on autohydrolysis; N: No effect on autohydrolysis; +: Band present; −: Band absent.

## Data Availability

The data supporting the findings of this manuscript are available from the corresponding authors upon reasonable request.

## Supplementary Materials

The following supporting information can be downloaded at: https://www.mdpi.com/article/10.3390/biom16050690/s1, Figure S1. Absorbance response curve of EmAGA toward gradient concentrations of Asp(pNA)-OH; Figure S2. Structural alignment of substrate binding regions between the EmAGA docking model (colored in blue) and the homologous crystal structure (PDB: 4R4Y, colored in gray).
