# Supplementary material for: Recharacterization of EmAGA, a Potential Candidate for Novel ALL Therapeutics
Source: Biomolecules. 2026 May 7;16(5):690. doi: 10.3390/biom16050690 (PMC13204146; doi:10.3390/biom16050690)
Supplement: Supplementary file 1 [file biomolecules-16-00690-s001.zip › biomolecules-4224055-supplementary.pdf]

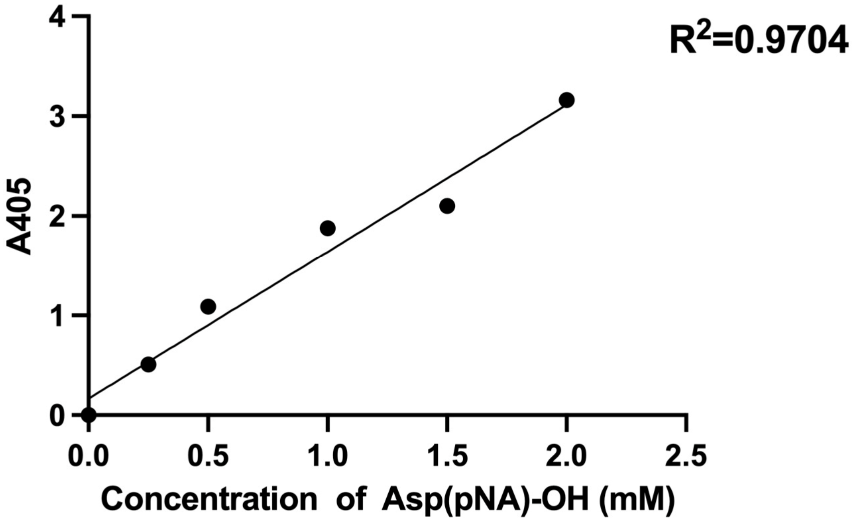

Figure S1. Absorbance response curve of EmAGA toward gradient concentrations of Asp(pNA)-OH. EmAGA (2 μg) was incubated with Asp(pNA)-OH at concentrations ranging from 0 to 2.0 mM, and the absorbance was determined at 405 nm. The dotted line represents the linear fitting curve with R^2=0.9704.

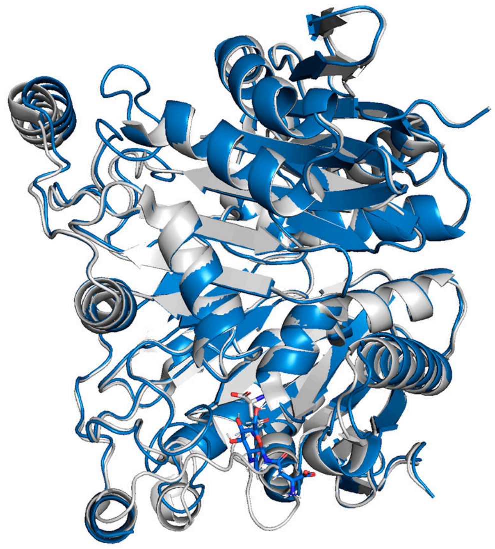

Figure S2. Structural alignment of substrate binding regions between the EmAGA docking model (colored in blue) and the homologous crystal structure (PDB: 4R4Y, colored in grey)
